# The effect of priming and duration of oestradiol benzoate treatment before progesterone administration on embryo development and survival in anestrous recipient mares

**DOI:** 10.1111/rda.14220

**Published:** 2022-08-08

**Authors:** Pedro S. Oquendo, Elisa S. M. Silva, Fabiana M. G. Oquendo, Juan Cuervo‐Arango, Marcelo E. Beletti

**Affiliations:** ^1^ Faculty of Veterinary Medicine Federal University of Uberlandia Uberlandia Brazil; ^2^ Departamento de Medicina y Cirugía Animal, Facultad de Veterinaria Universidad Cardenal Herrera‐CEU, CEU Universities Valencia Spain; ^3^ Institute of Biomedical Sciences Federal University of Uberlandia Uberlandia Brazil

**Keywords:** anestrous recipient, embryo transfer, oestradiol treatment, mare

## Abstract

The effect of three different hormonal protocols to prepare anestrous recipient mares on embryo survival was evaluated. The first group consisted of only progesterone administration (NE) 4 days before embryo transfer, while the recipients from the other two groups received a single administration of 2.5 mg of oestradiol benzoate (SE) 2 days earlier or 8 mg of oestradiol split in increasing doses for 5 consecutive days (LE) ending 3 days before progesterone treatment. The likelihood of recovering an embryo 2 days after transfer was 46.1% (6/13), 62.5% (5/8) and 85.7% (6/7) for recipient mares from the no oestrus, short and long oestrous groups respectively (*p* = .09). In conclusion, the presence and duration of oestradiol treatment before progesterone administration tended to influence the embryo survival in anestrous recipients 2 days after transfer. The surviving embryos recovered from the three different groups of recipients did not show any difference in size and morphology.

## 1 INTRODUCTION

Embryo transfer (ET) is a common practice in equine‐assisted reproduction programs. Mares are seasonal polyoestrous long day breeders and, the majority of them, undergo a period of winter anestrus (Aurich, 2011). Therefore, the use of hormonal protocols in seasonal anestrous mares is a crucial tool to increase the efficiency of ET clinical programs since it allows the possibility of transferring embryos outside the breeding season.

Although progesterone alone can be used to prepare anestrous recipient mares to receive embryos and maintain the pregnancy to term (Hinrichs et al., 1987), pre‐exposure to oestradiol before progesterone dominance appears to improve the endometrial environment to increase embryo survival (Cuervo‐Arango, Claes, de Ruijter‐Villani, et al., 2018; Silva et al., 2019).

Furthermore, the duration of oestrus before ET has been correlated positively with the fertility of cyclic recipients (Cuervo‐Arango, Claes, de Ruijter‐Villani, et al., 2018). In anestrous recipients, a longer exposure of the endometrium to oestrogens before progesterone administration resulted in greater expression of uterocalin (Silva et al., 2019), a protein which has been shown to be relevant for embryo nutrition (Crossett et al., 1998). However, it is unknown to which extent the presence and durations of oestrogen treatment affects embryo development and viability after ET in acyclic recipients. Therefore, the present study aimed to evaluate the influence of different durations of oestrogen treatment, or its absence, before P4 administration, on embryo development and survival. It was hypothesized that the presence and longer duration of oestrogen treatment before progesterone administration would increase (1) embryo survival within 48 h of transfer; and (2) growth rate of surviving embryos by 48 h of transfer.

## 2 MATERIALS AND METHODS

### 2.1 Animals

A total of 43 crossbred mares were used, 22 donors and 21 recipients, between five and 15 years of age and weighing between 350 and 450 kg. The experiment took place over two consecutive years, from July to October 2019 and 2020, in an equine breeding centre in Uberlândia—Minas Gerais—Brazil. Donor and recipient mares were selected based on ovarian activity. Donors were included in the study when they were cyclic: presence of regular follicular growth followed by signs of oestrus and ovulation. Only seasonal anestrous recipient mares were enrolled in the study: follicles <20 mm and absence of a CL for at least 21 days before being enrolled in the study.

### 2.2 Experimental design and groups

Donor mares were inseminated with a stallion of proven fertility. Embryo flushing was performed 8 days after donor's ovulation. Embryo diameter and grade were recorded according to McKinnon and Squires (1988). Only grade I and II expanded blastocysts were used for the study.

**FIGURE 1:**
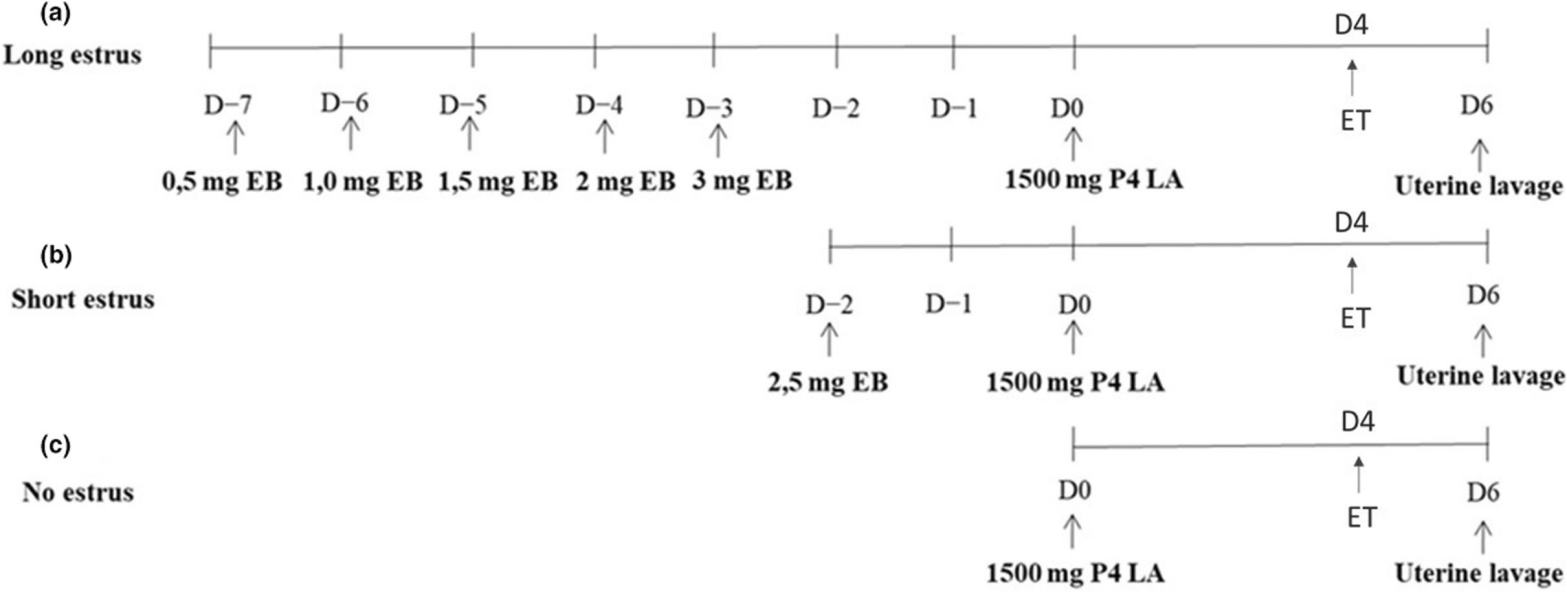
Schematic representation of hormonal treatments carried out in non‐cyclic mares in the three groups of recipients: (a) long oestrus, (b) short oestrus and (c) no oestrus. EB, oestradiol benzoate; ET, embryo transfer; P4 LA, long‐acting progesterone

Embryo recipients were divided into three groups, according to the hormonal protocol received: (I) Long Oestrus (LE), in which the mares were treated with a total of 8 mg of oestradiol benzoate (EB) in increasing doses for 5 days consecutively, and 72 h later received 1500 mg of long acting progesterone (LA P4) (Figure 1a); (II) Short Oestrus (SE), which received a single dose of 2.5 mg of EB and, 48 h later, 1500 mg of LA P4 (Figure 1b); and (III) No oestrus (NE), which received a single administration of 1500 mg of LA P4, without previous administration of EB (Figure 1c). Hormonal treatment with EB started 1 day before ovulation induction in donors in the LE group, 2 days after the donor ovulation in the SE group and 4 days after the donor ovulation in the NE group, so that all recipient groups received a D8 embryo on the fourth day after LA P4 administration (D4). Embryos were transferred into recipients using a modified Wilsher's technique as described previously (Cuervo‐Arango, Claes, & Stout, 2018). To evaluate embryo survival and viability, embryo recovery was attempted 2 days after transfer by uterine lavage (D6; Figure 1). When recovery was positive, the embryos were evaluated for grade and size.

### 2.3 Statistical analyses

To investigate the effect of the hormonal protocol used to prepare the recipient mares on embryo survival, a binary logistic regression model was used. The effect of the hormonal protocol on embryo growth rate was determined by a general linear model of variance with a repeated statement for sequential observations. A probability of *p* < .05 indicates that a difference was significant, whereas probabilities between *p* > .05 and *p* ≤ .1 indicates that a difference approached significance.

## 3 RESULTS

Overall, 43 inseminations of donor mares resulted in the recovery and transfer into recipient mares of 28 embryos. The regression model showed that the embryo survival tended to be influenced (*p =* .09) by the duration of oestrus. The year of the study or the donor age did not influence the embryo survival (*p* > .1). The likelihood of recovering an embryo 2 days after transfer was 46.1% (6/13), 62.5% (5/8) and 85.7% (6/7) for recipient mares from the NE, SE and LE groups respectively. The initial embryo diameters and those following embryo recovery for the three treatment groups are shown in Table 1. The embryo growth rate or grade 2 days after transfer was not influenced by the hormonal treatment group (*p* > .1).

**TABLE 1:** Mean embryo diameter ± SD at day 8 (before transfer) and day 10 (after embryo flushing and recovery) and mean increase in size by 48 h in long oestrus (LE), short oestrus (SE) and no oestrus (NE) groups

Group	Day 8 (μm)	Day 10 (μm)	Increase in size by 48 h
NE (*n* = 6)	753 ± 157.6	2509.6 ± 363.9	3.1 ± 1.02
SE (*n* = 5)	659.8 ± 169.6	2736.6 ± 463.1	4.2 ± 0.6
LE (*n* = 6)	601.5 ± 157.8	2587.2 ± 932.6	4.3 ± 1.03

## 4 DISCUSSION

Hypothesis 1 is partially (approached significance) substantiated by the results of this study. The positive trend between the increasing duration in the exposure of the endometrium to oestrogens (from absence to 7 days) observed in the results of the present study are similar to those reported in cyclic mares (Cuervo‐Arango, Claes, de Ruijter‐Villani, & Stout, 2018). Furthermore, the embryo survival observed in anestrous mares treated only with progesterone (6/13, 46%) was similar (3/7, 43%) to that reported by Hinrichs et al. (1987) using ovariectomized mares treated with progesterone only. Thus, it seems that about half of embryos do not need a uterine environment, which has been primed with oestrogens to survive and develop normally. However, the other half appear to quickly die in a suboptimal endometrial environment which has not been enriched by the action of oestrogen priming.

Although the duration of oestrus before ovulation in cyclic mares (Cuervo‐Arango, Claes, de Ruijter‐Villani, & Stout, 2018) and the duration of oestrogen treatment in anestrous mares before progesterone treatment (Silva et al., 2019) have been shown to influence the likelihood of pregnancy following ET and the endometrial gene expression profile, respectively, the results of this study failed to show any difference in embryo survival between SE and LE groups. It is likely that the power of the study was not enough to show a significant difference due to the relatively low number of embryos transferred in the SE and LE groups. In a previous study (Cuervo‐Arango, Claes, de Ruijter‐Villani, & Stout, 2018) using cyclic recipients, the pregnancy rate of recipients with a short oestrus (≤3 days) was 65%, about 20% lower than that of recipients with a longer oestrus (84%).

Hypothesis 2 of the study is rejected, since the embryo grade and growth rate of surviving embryos was not different amongst recipient groups. This finding may indicate that if the endometrial environment is not adequate to support embryo development following transfer, the embryo appears to die and disappear relatively quickly, at least within 48 h, before the embryo recovery was attempted.

## 5 CONCLUSIONS

The presence and duration of oestradiol treatment before progesterone administration tended to influence the embryo survival in anestrous recipients 2 days after transfer. The surviving embryos recovered from the three different groups of recipients did not show any difference in size and morphology 2 days after ET.

## AUTHOR CONTRIBUTIONS

PSO was responsible for data collection and manuscript writing, EMS contributed with experiment design, data analysis and manuscript writing. FMO assisted with data collection. JCA contributed with experimental design, statistical analysis and manuscript writing. MEB contributed with statistical analysis, manuscript writing and revision.

## CONFLICT OF INTEREST

None of the authors have any conflict of interest to declare.

## Data Availability

The data is availabe from the corresponding author upon request
